# Total cholesterol and all-cause mortality by sex and age: a prospective cohort study among 12.8 million adults

**DOI:** 10.1038/s41598-018-38461-y

**Published:** 2019-02-07

**Authors:** Sang-Wook Yi, Jee-Jeon Yi, Heechoul Ohrr

**Affiliations:** 1 Department of Preventive Medicine and Public Health, Catholic Kwandong University College of Medicine, Gangneung, 25601 Republic of Korea; 2 Institute for Clinical and Translational Research, Catholic Kwandong University College of Medicine, Gangneung, 25601 Republic of Korea; 3 Institute for Occupational and Environmental Health, Catholic Kwandong University, Gangneung, 25601 Republic of Korea; 4 Department of Preventive Medicine, Yonsei University College of Medicine, Seoul, 03722 Republic of Korea

## Abstract

It is unclear whether associations between total cholesterol (TC) levels and all-cause mortality and the optimal TC ranges for lowest mortality vary by sex and age. 12,815,006 Korean adults underwent routine health examinations during 2001–2004, and were followed until 2013. During follow-up, 694,423 individuals died. U-curve associations were found. In the TC ranges of 50–199 and 200–449 mg/dL, each 39 mg/dL (1 mmol/L) increase in TC was associated with 23% lower (95% CI:23%,24%) and 7% higher (6%,7%) mortality, respectively. In the age groups of 18–34, 35–44, 45–54, 55–64, 65–74, and 75–99 years, each 1 mmol/L higher TC increased mortality by 14%, 13%, 8%, 7%, 6%, and 3%, respectively (*P* < 0.001 for each age group), for TC ≥ 200 mg/dL, while the corresponding TC changes decreased mortality by 13%, 27%, 34%, 31%, 20%, and 13%, respectively, in the range < 200 mg/dL (*P* < 0.001 for each age group). TC had U-curve associations with mortality in each age-sex group. TC levels associated with lowest mortality were 210–249 mg/dL, except for men aged 18–34 years (180–219 mg/dL) and women aged 18–34 years (160–199 mg/dL) and 35–44 years (180–219 mg/dL). The inverse associations for TC < 200 mg/dL were stronger than the positive associations in the upper range.

## Introduction

Reduction of total cholesterol (TC) has been an integral part of public health campaigns, such as Healthy People 2020 in the US and Under 5 in Norway^1–3^, as well as cardiovascular disease (CVD) risk prediction models. This goal has primarily been supported by the success of statin trials showing that statin therapy reduced mortality from ischemic heart disease (IHD)^4,5^. “The lower, the better” cholesterol hypothesis has been accepted by many health professionals. However, the statin trials were mainly performed in persons at a high risk of heart disease, especially in men with manifest CVD, in whom heart disease mortality constituted approximately 50% of all deaths^6^.

Although disease-specific morbidity and mortality, such as IHD mortality, have their analytical merits, all-cause mortality is arguably the most important endpoint for patients or the general population when assessing risk factors and the effectiveness of a treatment or a public health intervention for life-threatening diseases^7^. The target TC levels for public health interventions in the general population should be determined after careful consideration of the levels associated with the lowest mortality in the general population. The associations of TC and all-cause mortality, however, have been relatively infrequently examined, and the associations have been inconsistent: positive linear^8^, inverse^9^, U-curve^10–12^, and reverse-L-curve^13,14^ associations have all been found. Moreover, cholesterol levels differ by sex and age^15–18^. It is unclear whether and to what extent the associations of cholesterol with mortality differ by sex and age^3,17^.

Through a large prospective cohort study among over 12 million participants, we examined whether the association between TC levels and all-cause mortality varied by sex and age, and estimated the sex- and age-specific levels of TC associated with the lowest mortality. Additionally, detailed estimates of the mean (and median) concentrations of TC according to sex and age are presented.

## Methods

### Study population and follow-up

Ninety-seven percent of the Korean population receives compulsory health insurance through the National Health Insurance Service (NHIS). The Korean Metabolic Risk Factor (KOMERIT) study included 12,845,017 NHIS beneficiaries 18–99 years of age who underwent routine health examinations from 2001 to 2004^19^. Persons (n = 26,136) with missing information on serum total cholesterol, fasting glucose, blood pressure, and body mass index (BMI) were excluded, as were 3,665 individuals with extreme anthropometric measures and another 210 with a missing date of the health examination. The final study population included 12,815,006 participants, who were followed until December 31, 2013 through the Resident Register of Korea. The authors were granted access to the anonymized data by the NHIS, without specific informed consent from the participants according to Korean law. This study was approved by the Institutional Review Board of Catholic Kwandong University with a waiver of informed consent. The Strengthening the Reporting of Observational Studies in Epidemiology (STROBE) checklist for cohort studies was used to guide the reporting of our study.

### Data collection

Serum TC and fasting glucose were assayed using enzymatic methods. Blood pressure was measured once in a seated position using a standard mercury sphygmomanometer, and the systolic blood pressure was measured as the first Korotkoff sound. Weight and height were measured to the nearest kilogram and centimeter, respectively^19^. BMI was calculated by weight in kilograms divided by the square of height in meters (kg/m^2^). Information on smoking history, alcohol use, and known heart disease or cancers was collected via a self-administered questionnaire. A standard protocol officially registered by the Korean government was applied for health examinations and data collection. External quality assessments of clinical chemistry were regularly performed^20^.

### Statistical analysis

Baseline TC concentrations were mainly categorized into 18 groups (mg/dL; <120, 120–129 to 270–279 in increments of 10, ≥280). The cholesterol category with the lowest mortality (220–229 mg/dL) in all participants was used as the reference. Three groups (<200 [reference, desirable], 200–239 [borderline high], and ≥240 [high]), defined according to the cut-points proposed by the National Cholesterol Education Program (NCEP) of US, were used in an additional analysis^21^. In the spline analysis, a restricted cubic spline transformation of TC with 5 knots (138, 170, 191, 213, and 260 mg/dL; 5th, 27.5th, 50th, 72.5th, and 95th percentiles in all participants) was used to evaluate non-linear associations.

The hazard ratios (HRs) for death were calculated using Cox proportional hazards models stratified by age (years) at baseline (18–24, 25–34, 35–44, 45–54, 55–64, 65–74, or 75–99). In the multivariable model, the following variables were adjusted for: age at baseline (continuous variable; within each age group), sex, smoking status (current smoker, former smoker, never smoker, or missing information), alcohol use frequency (none, 2 days/month-2 days/week, 3–7 days/week, or missing information), physical activity (at least once a week; yes or no), systolic blood pressure (<120, 120–139, or ≥140 mm Hg), fasting glucose (<100, 100–125, or ≥126 mg/dL), and BMI (<18.5, 18.5–24.9, 25–29.9, or ≥30 kg/m^2^).

The apparent optimal ranges of TC for survival were determined by a general inspection of the curvilinear association. Generally, the interval of 40 mg/dL (roughly 1 mmol/L) with the lowest risk (the lowest unweighted geometric mean of HRs in 4 consecutive TC categories in the categorical analysis, and in 5 consecutive 10-mg/dL TC levels in the spline analysis [for example, the points of 200, 210, 220, 230, 240 mg/dL]), were considered the optimal ranges.

Subgroup analyses by sex and age, as well as various categorical, spline, and linear analyses, served as sensitivity analyses. All p-values were 2-sided. All analyses used SAS version 9.4 (SAS Institute Inc., Cary, NC, USA).

### Ethics approval

This study was approved by the Institutional Review Board of Kwandong University (Gangneung, Republic of Korea).

## Results

During a mean 10.5 years of follow-up, 454,546 men and 239,877 women died. At baseline, the participants’ mean ± SD age was 44.4 ± 14.2 years, their mean TC level was 194.2 ± 49.0 mg/dL (Table 1), and 11.2% of participants had high TC levels (≥240 mg/dL). Individuals with higher TC levels were older and had higher levels of fasting glucose, systolic blood pressure, and BMI (Table 1). People with TC ≥240 mg/dL tended to be non-drinkers and were more likely to have comorbid heart disease, stroke, or cancer. The number of individuals was highest in the TC range of 180–189 mg/dL (Table 1, Supplementary Fig. 1).

**Table 1:** Participants’ characteristics according to total cholesterol categories.

Variables	Characteristics	Total	Desirable	Borderline	High
			<200 mg/dL	200–239 mg/dL	≥240 mg/dL
		N = 12,815,006	n = 7,633,687	n = 3,739,930	n = 1,441,389
Total cholesterol	mg/dL^a^	194.2 ± 49.0	169.1 ± 20.8	216.6 ± 11.1	268.9 ± 92.3
Age	years	44.4 ± 14.2	42.1 ± 14.3	46.9 ± 13.5	49.8 ± 13.0
Systolic blood pressure	mm Hg	124.1 ± 17.3	122.0 ± 16.6	126.3 ± 17.4	129.4 ± 18.2
Fasting serum glucose	mg/dL^b^	94.9 ± 31.0	92.6 ± 27.9	96.7 ± 30.6	102.7 ± 43.4
Body mass index	kg/m^2^	23.5 ± 3.2	22.9 ± 3.1	24.2 ± 3.1	24.8 ± 3.1
Sex	Women	7,292,064 (56.9)	4,327,185 (56.7)	2,178,135 (58.2)	786,744 (54.6)
	Men	5,522,942 (43.1)	3,306,502 (43.3)	1,561,795 (41.8)	654,645 (45.4)
Smoking status	Never smoker	3,653,334 (28.5)	2,195,184 (28.8)	1,063,607 (28.4)	394,543 (27.4)
	Past smoker	7,425,581 (57.9)	4,426,732 (58.0)	2,152,551 (57.6)	846,298 (58.7)
	Current smoker	1,099,436 (8.6)	619,146 (8.1)	347,798 (9.3)	132,492 (9.2)
	Missing	636,655 (5.0)	392,625 (5.1)	175,974 (4.7)	68,056 (4.7)
Alcohol use frequency, days	None	6,102,884 (47.6)	3,538,707 (46.4)	1,814,686 (48.5)	749,491 (52.0)
	2/month-2/week	4,980,284 (38.9)	3,080,461 (40.4)	1,409,936 (37.7)	489,887 (34.0)
	3–7/week	1,237,846 (9.7)	714,422 (9.4)	375,968 (10.1)	147,456 (10.2)
	Missing	493,992 (3.9)	300,097 (3.9)	139,340 (3.7)	54,555 (3.8)
Physical activity	≥1 times/week	5,158,300 (40.3)	3,028,392 (39.7)	1,545,436 (41.3)	584,472 (40.5)
History of heart disease	Yes	107,365 (0.8)	57,802 (0.8)	33,851 (0.9)	15,712 (1.1)
History of stroke	Yes	48,669 (0.4)	24,739 (0.3)	16,087 (0.4)	7,843 (0.5)
History of cancer	Yes	49,237 (0.4)	28,985 (0.4)	14,213 (0.4)	6,039 (0.4)
Age group, years	18–34	3,729,737 (29.1)	2,739,434 (35.9)	785,022 (21.0)	205,281 (14.2)
	35–44	3,256,771 (25.4)	1,977,540 (25.9)	957,884 (25.6)	321,347 (22.3)
	45–54	2,717,047 (21.2)	1,388,813 (18.2)	931,679 (24.9)	396,555 (27.5)
	55–64	1,863,363 (14.5)	887,619 (11.6)	655,108 (17.5)	320,636 (22.2)
	65–74	994,911 (7.8)	502,150 (6.6)	331,674 (8.9)	161,087 (11.2)
	75–99	253,177 (2.0)	138,131 (1.8)	78,563 (2.1)	36,483 (2.5)
Total cholesterol, mg/dL	<120	122,981 (1.0)	122,981 (1.6)	0 (0.0)	0 (0.0)
	120–129	185,401 (1.4)	185,401 (2.4)	0 (0.0)	0 (0.0)
	130–139	402,908 (3.1)	402,908 (5.3)	0 (0.0)	0 (0.0)
	140–149	642,593 (5.0)	642,593 (8.4)	0 (0.0)	0 (0.0)
	150–159	935,938 (7.3)	935,938 (12.3)	0 (0.0)	0 (0.0)
	160–169	1,189,951 (9.3)	1,189,951 (15.6)	0 (0.0)	0 (0.0)
	170–179	1,349,594 (10.5)	1,349,594 (17.7)	0 (0.0)	0 (0.0)
	180–189	1,418,033 (11.1)	1,418,033 (18.6)	0 (0.0)	0 (0.0)
	190–199	1,386,288 (10.8)	1,386,288 (18.2)	0 (0.0)	0 (0.0)
	200–209	1,227,973 (9.6)	0 (0.0)	1,227,973 (32.8)	0 (0.0)
	210–219	1,037,227 (8.1)	0 (0.0)	1,037,227 (27.7)	0 (0.0)
	220–229	850,522 (6.6)	0 (0.0)	850,522 (22.7)	0 (0.0)
	230–239	624,208 (4.9)	0 (0.0)	624,208 (16.7)	0 (0.0)
	240–249	456,833 (3.6)	0 (0.0)	0 (0.0)	456,833 (31.7)
	250–259	342,597 (2.7)	0 (0.0)	0 (0.0)	342,597 (23.8)
	260–269	224,887 (1.8)	0 (0.0)	0 (0.0)	224,887 (15.6)
	270–279	136,285 (1.1)	0 (0.0)	0 (0.0)	136,285 (9.5)
	≥280	280,787 (2.2)	0 (0.0)	0 (0.0)	280,787 (19.5)

Data are expressed as mean ± SD or n (%).

The *p* values, which were calculated by the chi-square test and 1-way ANOVA across the cholesterol groups, were <0.001 for each variable.

^a^To convert total cholesterol from mg/dL to mmol/L, multiply by 0.02586.

^b^To convert glucose from mg/dL to mmol/L, multiply by 0.0555.

SD, standard deviation; ANOVA, analysis of variance.

### TC concentrations according to sex and age

**Figure 1:**
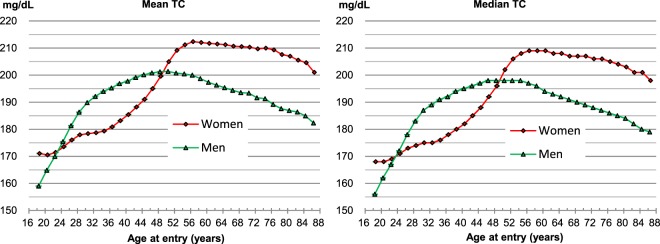
Mean and median concentrations of total cholesterol. To convert cholesterol from mg/dL to mmol/L, multiply by 0.02586.

Men had on average higher TC levels than women between 24–25 to 48–49 years old, while women had higher levels than men in the age ranges of 18–23 years and ≥50 years (Fig. 1, Supplementary Table S1). Among men, the mean TC levels increased from 159.0 mg/dL at 18–19 years to a maximum of 201.4 mg/dL at 50–51 years, and among women, the mean levels increased from 170.5 mg/dL at 20–21 years to a maximum of 212.4 mg/dL at 56–57 years. The decrease in TC levels after the peak values were reached was greater in men than in women. The gradient of increase in TC levels with age was steepest from 18–19 to 28–29 years in men, while it was steepest from 44–45 to 52–53 years in women (Fig. 1).

### Associations between total cholesterol and mortality

**Figure 2:**
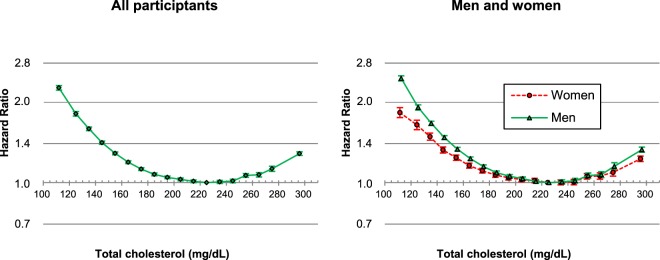
Age-adjusted hazard ratios associated with 18 total cholesterol (TC) categories, according to sex. TC categories (mg/dL: <120, 120–129 to 270–279 by 10, ≥280, 220–229 as reference). The midpoint was used as a representative value for each TC category, except for both ends (112 and 296), for which the median of all participants was used. *Hazard ratios and 95% confidence intervals were calculated using Cox hazards models stratified by baseline age (years: 18–24, 25–34, 35–44, 45–54, 55–64, 65–74, 75–84, 85–99). Age at baseline was adjusted as a continuous variable within each age group. To convert TC from mg/dL to mmol/L, multiply by 0.02586.

**Figure 3:**
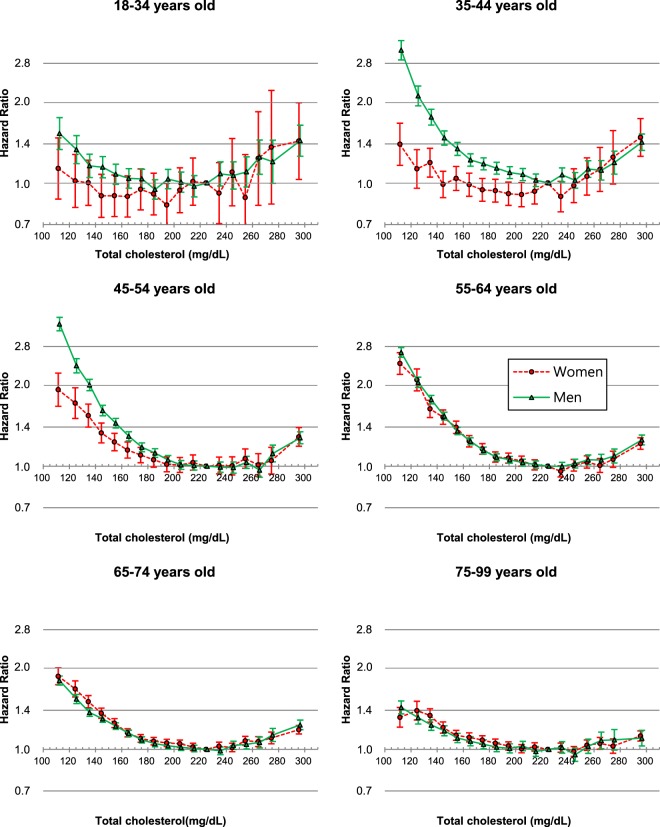
Hazard ratios^*^ associated with 18 total cholesterol (TC) categories for mortality by sex and age. TC categories (mg/dL: <120, 120–129 to 270–279 by 10, ≥280, 220–229 as reference). The midpoint was used as a representative value for each TC category, except for both ends (112 and 296), for which the median of all participants was used. *Hazard ratios and 95% confidence intervals were calculated using Cox hazards models stratified by baseline age (years: 18–24, 25–34, 35–44, 45–54, 55–64, 65–74, 75–84, 85–99), after adjustment for age at baseline (continuous variable), smoking status, alcohol use, physical activity, known history of heart disease, stroke, or cancer, body-mass index, systolic blood pressure, and fasting glucose levels. To convert TC from mg/dL to mmol/L, multiply by 0.02586.

U-curve associations between TC levels and mortality were found in both men and women (Fig. 2). The TC range associated with the lowest mortality was 210–249 mg/dL (Supplementary Table S2). When age was further considered, U-curve associations were observed regardless of sex or age (Fig. 3), and the optimal TC range for survival was 210–249 mg/dL for each age-sex group, except for men at 18–34 years (180–219 mg/dL) and for women at 18–34 years (160–199 mg/dL) and at 35–44 years (180–219 mg/dL) (Supplementary Table S3).

**Figure 4:**
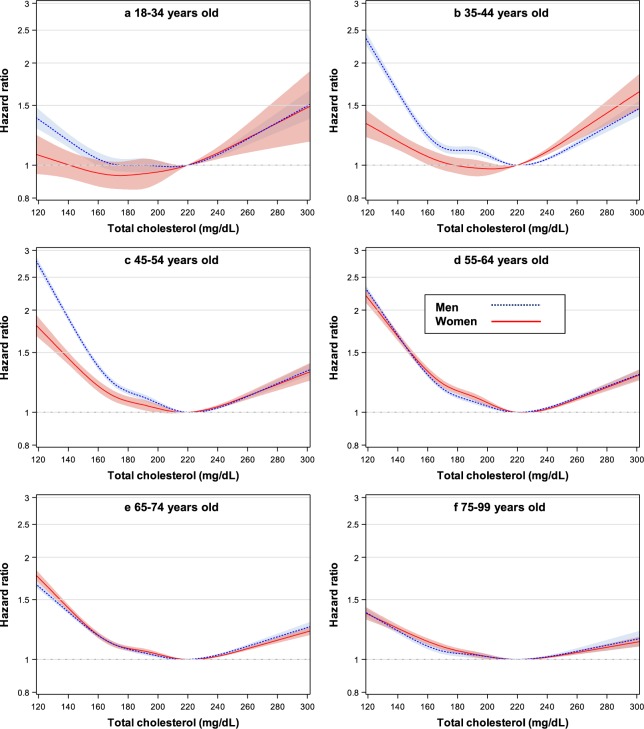
Hazard ratios^*^ using spline transformed total cholesterol (TC) levels for mortality by sex and age. 5 knots (138, 170, 191, 213, and 260 mg/dL) were used with 220 mg/dL as reference. *Hazard ratios and 95% confidence intervals were calculated using Cox hazards models with the same method as in Fig. 3. To convert TC from mg/dL to mmol/L, multiply by 0.02586.

In the spline analysis (Fig. 4, Supplementary Fig. 2), the TC ranges associated with the lowest mortality were approximately 200–240 mg/dL, except for men at 18–34 years (approximately 180–220 mg/dL) and for women at 18–34 years (approximately 160–200 mg/dL) and at 35–44 years (approximately 180–220 mg/dL).

**Figure 5:**
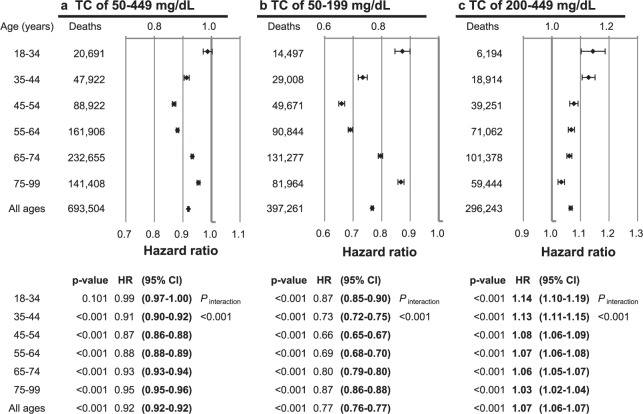
Hazard ratios^*^ per each 39 mg/dL (1 mmol/L) increase in total cholesterol (TC), according to TC range and age. *Hazard ratios and 95% confidence intervals were calculated using Cox hazards models with the same method as in Fig. 3. To convert TC from mg/dL to mmol/L, multiply by 0.02586.

When assuming linear associations for TC levels of 50–449, 50–199, and 200–449 mg/dL, each 39 mg/dL (1 mmol/L) increase in TC was associated with 8% lower (HR = 0.92, 95% CI = 0.917–0.922), 23% lower (HR = 0.77, 95% CI = 0.76–0.77), and 7% higher (HR = 1.07, 95% CI = 1.06–1.07) mortality, respectively (Fig. 5).

At cholesterol levels <200 mg/dL (Table 2), inverse associations were the strongest in men aged 45–54 years and women aged 55–64 years, the age group with the highest mean TC level in both sexes (*P*_interaction_ [age] <0.001). At cholesterol levels ≥200 mg/dL, the HRs per 39 mg/dL (1 mmol/L) higher levels were highest in the youngest groups (aged 18–44 years), and lowest in the oldest group (aged 75–99) years in both sexes (*P*_interaction_ [age] <0.001).

**Table 2:** HRs^a^ per 39 mg/dL (1 mmol/L) TC increase according to sex, age, and TC range.

TC range,	Age group,	Men			Women			p value for interaction between sexes
mg/dL (mmol/L)	years	No. of deaths	p-value	HR (95% CI)	No. of deaths	p-value	HR (95% CI)	
50–449	All ages	453,959	<0.001	0.90 (0.89–0.90)	239,545	<0.001	0.95 (0.95–0.96)	<0.001
	18–34	16,111	0.098	0.98 (0.97–1.00)	4,580	0.231	1.02 (0.99–1.06)	0.071
	35–44	37,382	<0.001	0.90 (0.89–0.91)	10,540	0.468	0.99 (0.97–1.01)	<0.001
	45–54	67,370	<0.001	0.85 (0.84–0.85)	21,552	<0.001	0.95 (0.93–0.96)	<0.001
	55–64	116,119	<0.001	0.86 (0.86–0.87)	45,787	<0.001	0.92 (0.92–0.93)	<0.001
	65–74	144,609	<0.001	0.92 (0.91–0.92)	88,046	<0.001	0.96 (0.95–0.96)	<0.001
	75–99	72,368	<0.001	0.94 (0.93–0.95)	69,040	<0.001	0.97 (0.96–0.97)	<0.001
50–199	All ages	283,239	<0.001	0.75 (0.74–0.75)	114,022	<0.001	0.81 (0.80–0.82)	<0.001
	18–34	10,889	<0.001	0.87 (0.84–0.90)	3,608	0.009	0.92 (0.87–0.98)	0.079
	35–44	21,936	<0.001	0.70 (0.68–0.72)	7,072	<0.001	0.86 (0.83–0.90)	<0.001
	45–54	38,832	<0.001	0.64 (0.63–0.65)	10,839	<0.001	0.77 (0.74–0.80)	<0.001
	55–64	70,570	<0.001	0.68 (0.67–0.69)	20,274	<0.001	0.72 (0.70–0.74)	<0.001
	65–74	91,938	<0.001	0.80 (0.79–0.81)	39,339	<0.001	0.80 (0.78–0.81)	0.889
	75–99	49,074	<0.001	0.86 (0.85–0.88)	32,890	<0.001	0.87 (0.86–0.89)	0.356
200–449	All ages	170,720	<0.001	1.07 (1.07–1.08)	125,523	<0.001	1.06 (1.05–1.06)	<0.001
	18–34	5,222	<0.001	1.15 (1.10–1.19)	972	0.006	1.14 (1.04–1.26)	0.953
	35–44	15,446	<0.001	1.12 (1.09–1.14)	3,468	<0.001	1.19 (1.14–1.25)	0.017
	45–54	28,538	<0.001	1.08 (1.06–1.09)	10,713	<0.001	1.08 (1.05–1.11)	0.790
	55–64	45,549	<0.001	1.07 (1.05–1.08)	25,513	<0.001	1.06 (1.05–1.08)	0.761
	65–74	52,671	<0.001	1.07 (1.05–1.08)	48,707	<0.001	1.05 (1.04–1.07)	0.143
	75–99	23,294	0.008	1.03 (1.01–1.05)	36,150	<0.001	1.03 (1.02–1.05)	0.535

CI, confidence interval; HR, hazard ratio; TC, total cholesterol.

^a^HRs were calculated by Cox models stratified by age (baseline age, years: 18–24, 25–34, 35–44, 45–54, 55–64, 65–74, 75–84, 85–99), after adjustement for age at baseline, sex (if applicable), smoking status, alcohol use, physical activity, known history of heart disease, stroke, or cancer, body mass index, systolic blood pressure, and total cholesterol.

To convert glucose from mg/dL to mmol/L, multiply by 0.02586.

The associations were modestly stronger in men than in women at TC levels of 50–449, 50–199, and 200–449 mg/dL (*P*_interaction_ [sex] <0.001 in each TC range), when all ages were combined. At cholesterol levels <200 mg/dL, men had stronger inverse associations than women in age groups <65 years.

### Associations across standard classifications of TC

Compared to the desirable levels of <200 mg/dL (Supplementary Table S3), borderline high levels of 200–239 mg/dL were associated with a lower risk of mortality in each age-sex group except for women aged 18–34 years, while high levels of ≥240 mg/dL were associated with a decreased risk in both sexes and each age group except for women aged 18–44 years and men aged 18–34 years, in whom high levels were associated with increased mortality.

## Discussion

A U-shaped relationship between TC and mortality was observed in each age-sex group. TC levels associated with the lowest mortality were 210–249 mg/dL in both sexes in all age groups, except for the youngest groups of men, aged 18–34 years (180–219 mg/dL), and women aged 18–34 years (160–199 mg/dL) and 35–44 years (180–219 mg/dL). At TC levels of 50–199 and 200–449 mg/dL, each 39 mg/dL (1 mmol/L) increase in TC was associated with 23% lower (95% CI = 23–24%) and 7% higher (6–7%) mortality, respectively. Inverse associations in the lower TC range were strongest at the ages for which the mean TC levels were highest (men aged 45–54 years and women aged 55–64 years), while positive associations in the upper TC range were strongest in the youngest ages (<45 years) in both sexes. Both the inverse associations in the lower TC range and the positive associations in the upper TC range weakened with advancing age beyond the ages with the strongest associations.

Previous cohort studies have reported inconsistent results on the shape of associations between TC and all-cause mortality, including positive linear, inverse, U-curve, and reverse-L-curve (or reverse-J-curve) associations^3,8–11,13,14,17,22^. Some previous studies suggested different shapes of associations by sex and age^3,17^. The associationbetween TC and mortality was substantially modified by age and, to a lesser degree, by sex, in our study. Our study clearly demonstrated that the shape of association is a U-curve in each sex and each age group, including those aged 75–99 years (mean age: 79.0 years), which constituted 154,321, 80,776, and 18,080 elderly people aged 75–79, 80–84, and ≥85 years, respectively. Considering the weaker effect size associated with high TC with advancing age in the elderly, it is no surprise that previous studies conducted mainly in elderly populations found generally inverse or reverse-L-curve associations^13,14^. Additionally, the previously reported positive associations in younger adults^8^, may be explained by the stronger positive associations and lower optimal range in younger ages observed in our study, combined with the higher TC concentrations and larger proportions of morbidity and mortality from heart diseases in Western populations.

The NCEP experts classified TC levels into 3 categories: <200, 200–239, and ≥240 mg/dL, as desirable, borderline high, and high levels, respectively, mainly based on the association between TC and IHD^21^. In the current study, however, TC levels of 210–249 mg/dL and approximately 200–240 mg/dL were associated with the lowest mortality in the categorical and spline analyses, respectively. Our study suggested that the optimal ranges for overall survival are higher than that those for IHD. Similarly, a higher optimal range for overall survival than for IHD mortality has also been reported for BMI^23^. In contrast, the optimal ranges for all-cause mortality and IHD mortality were similar for fasting glucose and blood pressure^24–27^. Cholesterol levels might be a marker of general health, rather than a marker specific for CVD^28^. Even within CVD subtypes, TC ranges associated with lowest risk have not been consistent. For example, for stroke, TC levels <200 mg/dL were not associated with the lowest mortality in prospective cohort studies^29,30^, and randomized trials have not provided clear evidence of whether lipid-lowering therapies, including statins, reduce stroke mortality^6,31^. Hemorrhagic stroke, respiratory diseases (especially chronic obstructive pulmonary disease), digestive diseases (especially liver disease), and several cancers have been suggested to be associated with lower TC levels^10,30,32–34^; thus, the ranges associated with lowest risk might be even higher for these diseases than those for all-cause mortality. However, we could not examine whether the associations differed by cause of death, due to data unavailability.

Reverse causality has been suggested as an explanation of higher mortality associated with low cholesterol levels. However, a long term follow-up study in a Japanese-American population showed that individuals with low cholesterol levels maintained over a 20-year period had the worst all-cause mortality, and concluded that reverse causality was unlikely to account for the higher mortality associated with low cholesterol entirely^14^.

Lower optimal ranges for survival at younger ages than at older ages have also been observed for BMI^19^, whereas consistent ranges have been found regardless of sex and age for blood pressure and fasting glucose^26,27,29^. Whether different proportions of cause-specific mortality by age lead to the lower optimal range at younger ages needs to be investigated.

The sex- and age- specific levels of TC in the current study of Koreans were lower than those reported in other high-income countries, including Japan, England, and the US^15–17,35,36^. The distribution of TC levels by sex and age, however, were generally similar to those of other regional and ethnic populations, although detailed information is not always available. TC levels peaked at 50–51 years in men and at 56–57 years in women, and after the peak age, the levels decreased more rapidly in men than in women. The crossover point of the mean TC levels between sexes occurred at the age of 50–51 years, exactly at the median age of menopause^37^. The steep decline in estrogen corresponds well to the sharp increase in TC in women that was observed around the time of menopause in the current study.

Randomized trials have provided evidence that statin therapy may lower the overall mortality risk in persons with increased cardiovascular risk, mostly due to the reduction of mortality from heart disease^5,6^. The evidence, however, may not be definitive enough to claim that “the lower the cholesterol, the better” for all-cause mortality reduction in the general population with relatively low heart disease risk^38^.

The current cholesterol guidelines are heavily based on heart disease risk and recommend a TC range of <200 mg/dL as desirable. TC range <200 mg/dL, however, may not be necessarily a sign of good health when other diseases are considered. The diseases associated with lower TC levels and potential mechanisms have not been conclusively identified. Since the inverse associations in lower TC range were stronger than the positive associations in upper TC range, identification of diseases associated with lower TC levels and further understanding of the mechanisms of such associations may help improve health outcomes in the general population. Pending more research for clarification, careful evaluation and management might increase the chance of preventing and diagnosing potentially life-threatening diseases at an earlier stage in adults with low TC levels.

A very large number of participants, the prospective nature of the study, and complete follow-up for death are clear strengths of this study. Another major strength is that the study participants were ethnically homogeneous and lived in a similar environment covered by the same health care system. Another strength is that this study estimated mortality risk associated with TC levels down to below 120 mg/dL. However, there are limitations. First, the use of lipid-lowering medication was unaccounted for. The risk associated with high cholesterol might have been underestimated. However, in Korea, IHD mortality accounted for only approximately 5% of all-cause mortality, and only 10% of people with hypercholesterolemia received lipid-lowering therapy^39^. Therefore, the impact of not considering medication use is likely to be modest, and the TC levels in this study generally reflect levels without lipid-lowering medications. Additionally, this study could not determine whether statin-induced low cholesterol increases mortality. Second, other lipid measures, such as low-density lipoprotein and high-density lipoprotein cholesterol levels, were unavailable. Recent dyslipidemia management guidelines are more closely focused on these sub-fractions of cholesterol, so the direct application of our findings to individual patient care might be somewhat limited. Further study is needed to determine the sex- and age-specific associations of cholesterol fractions. Third, information on cause-specific mortality was not available. Fourth, the generalizability of our findings may be affected by the fact that the study participants were homogeneously Korean. The U-curve associations may be generalized to other ethnic populations, since the shape of the associations was generally the same for each sex and each age group, despite their varying cardiometabolic risk profiles. However, some results, such as the magnitude of relative risk associated with TC and the TC range associated with the lowest mortality, may vary by ethnic groups with different distributions of cause-specific mortality and dyslipidemia-related healthcare utilization.

In conclusion, U-curve relationships between TC and mortality were found, regardless of sex and age. TC ranges associated with the lowest mortality were 210–249 mg/dL in each sex-age subgroup, except for the youngest groups of men, aged 18–34 years (180–219 mg/dL), and women aged 18–34 years (160–199 mg/dL) and 35–44 years (180–219 mg/dL). Inverse associations in the range <200 mg/dL were more than 3-fold stronger than positive associations for cholesterol levels ≥200 mg/dL, except for the youngest adults. Positive associations in the upper TC range were strongest for youngest adults and weakened with advancing age. TC levels <200 mg/dL may not necessarily be a sign of good health. Identification and proper management of diseases associated with lower TC levels might improve survival.

## Supplementary information

Supplementary Figure and Table

## Data Availability

The data supporting the findings of this study are available from the NHIS, but restrictions apply to the availability of these data, which were used under license for the current study; therefore, the data are not publicly accessible.
