# Supplementary material for: Total cholesterol and all-cause mortality by sex and age: a prospective cohort study among 12.8 million adults
Source: Sci Rep. 2019 Feb 7;9:1596. doi: 10.1038/s41598-018-38461-y (PMC6367420; doi:10.1038/s41598-018-38461-y)
Supplement: Supplementary file 1 — Supplementary Figure and Table [file 41598_2018_38461_MOESM1_ESM.pdf]

Supplemental Digital Contents

Total cholesterol and all-cause mortality by sex and age: a prospective cohort study among 12.8 million adults

Sang-Wook Yi, MD, PhD, Department of Preventive Medicine and Public Health, Catholic Kwandong University College of Medicine, Republic of Korea; **Jee-Jeon Yi**, PhD, Institute for Occupational and Environmental Health, Catholic Kwandong University, Republic of Korea; **Heechoul Ohrr**, MD, PhD, Department of Preventive Medicine, Yonsei University College of Medicine, Republic of Korea

Correspondence: Sang-Wook Yi, MD, PhD, Department of Preventive Medicine and Public Health, Catholic Kwandong University College of Medicine, Bumil-ro 579, Gangneung, Gangwon-do, 25601, Republic of Korea; Tel: +82-33-649-7468; Fax +82-33-641-1074. e-mail: flyhigh@cku.ac.kr

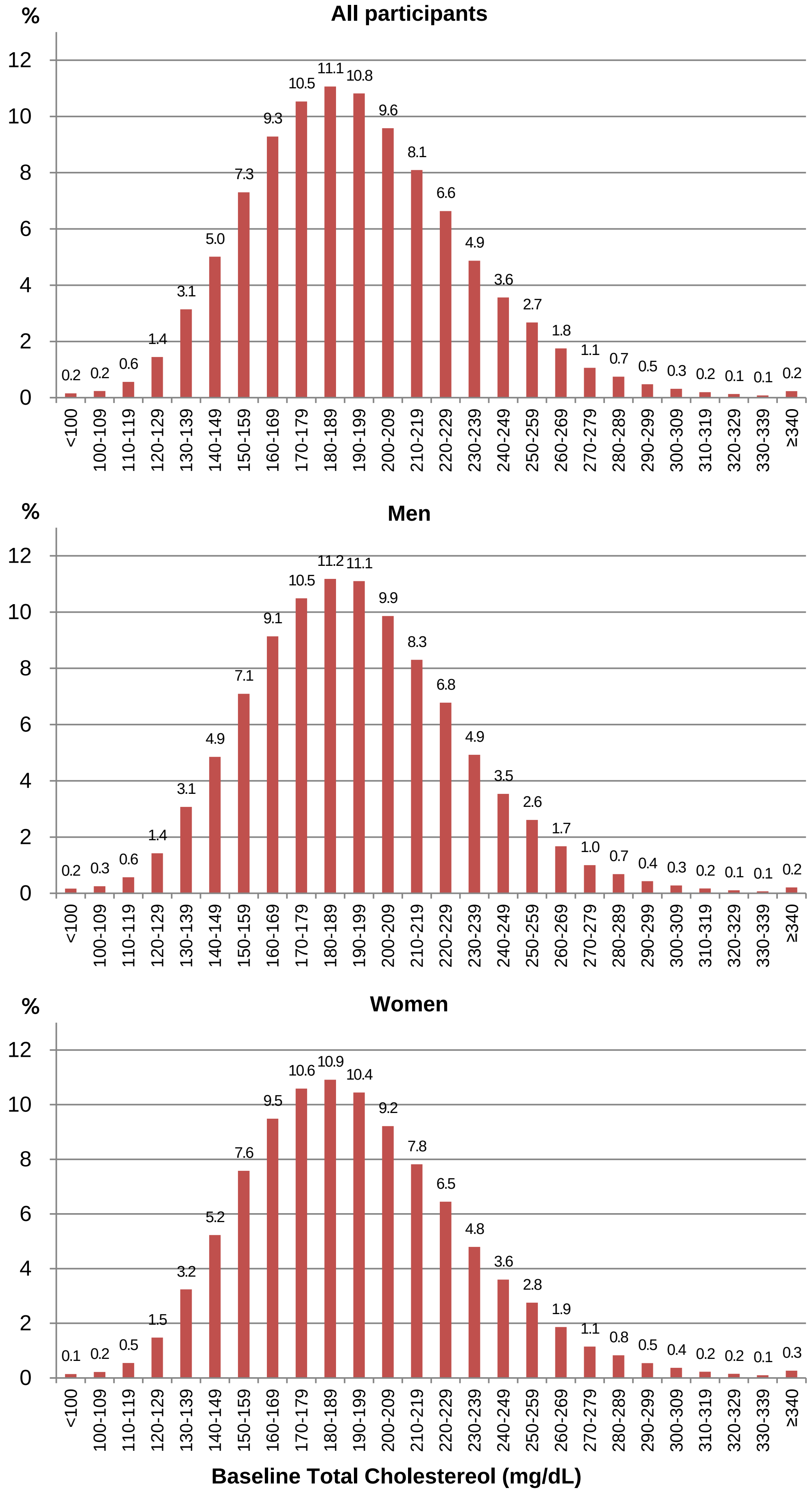

Figure S1. Distribution of total concentration in Korean adults.
To convert cholesterol from mg/dL to mmol/L, multiply by 0.02586.

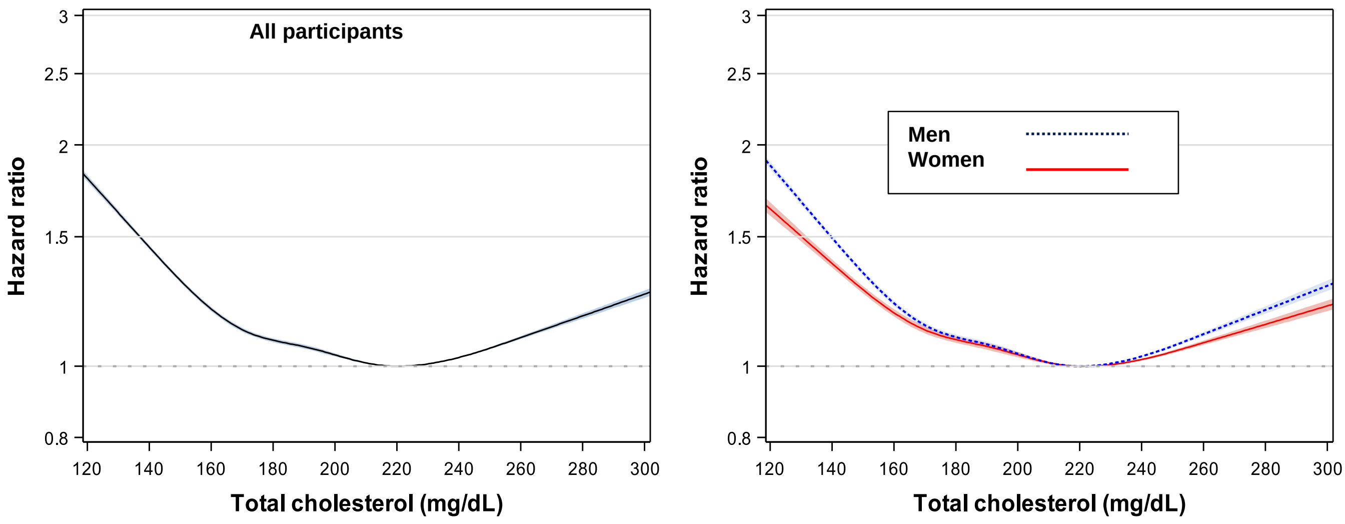

Figure S2. Hazard ratios[a] for all-cause mortality according to sex by restricted cubic splines of total cholesterol with five knots (138, 170, 191, 213, and 260 mg/dL) and 220 mg/dL as a reference

[a]Hazard ratios and 95% confidence intervals were calculated using Cox hazards models after adjustment for age at baseline (continuous variable), sex (when applicable), smoking status, alcohol use, physical activity, known history of heart disease, stroke, or cancer, body-mass index, systolic blood pressure, and fasting glucose levels. To convert cholesterol from mg/dL to mmol/L, multiply by 0.02586.

Table S1. Mean and median of total cholesterol (mg/dL) (Including numerical version of Figure 1).

Age,	Men and women				Men				Women				Sex difference[a]	
years	n	Median	Mean	(SD)	n	Median	Mean	(SD)	n	Median	Mean	(SD)	Median	Mean
18-99	12,815,006	191	194.2	(49.0)	7,292,064	191	194.1	(47.4)	5,522,942	190	194.3	(51.1)	1	-0.2
18-19	50,235	166	168.5	(32.5)	10,472	156	159.0	(29.2)	39,763	168	171.1	(32.9)	-12	-12.0
20-21	168,383	166	168.6	(34.5)	56,062	162	164.8	(35.5)	112,321	168	170.5	(33.8)	-6	-5.7
22-23	327,931	168	170.9	(37.8)	112,041	167	169.9	(39.4)	215,890	169	171.4	(36.9)	-2	-1.5
24-25	496,965	171	174.3	(38.5)	206,778	172	175.4	(39.4)	290,187	171	173.5	(37.8)	1	1.9
26-27	566,542	176	179.0	(40.6)	323,129	178	181.3	(41.8)	243,413	173	176.0	(38.8)	5	5.3
28-29	620,505	180	183.8	(42.0)	432,261	183	186.3	(42.8)	188,244	174	177.9	(39.6)	9	8.4
30-31	610,405	184	187.0	(41.6)	458,754	187	189.8	(41.8)	151,651	175	178.4	(39.9)	12	11.4
32-33	587,101	186	189.2	(42.9)	456,532	189	192.1	(43.1)	130,569	175	178.7	(40.7)	14	13.4
34-35	544,672	187	190.7	(43.0)	424,512	191	194.0	(43.8)	120,160	176	179.3	(37.9)	15	14.6
36-37	508,630	189	192.0	(42.6)	394,652	192	195.2	(43.2)	113,978	178	180.9	(38.3)	14	14.4
38-39	489,834	191	193.7	(43.5)	375,014	194	196.9	(43.8)	114,820	180	183.2	(40.9)	14	13.7
40-41	771,454	189	192.3	(45.0)	430,586	195	197.8	(44.9)	340,868	182	185.4	(44.3)	13	12.3
42-43	750,228	191	194.3	(47.3)	420,327	196	199.1	(47.3)	329,901	185	188.2	(46.5)	11	10.9
44-45	693,916	193	196.0	(47.8)	379,106	197	200.1	(47.3)	314,810	188	191.1	(48.0)	9	9.1
46-47	638,709	195	198.1	(48.4)	343,520	198	200.8	(45.9)	295,189	192	195.0	(51.0)	6	5.8
48-49	572,787	197	200.4	(48.8)	298,490	198	201.2	(47.4)	274,297	196	199.6	(50.3)	2	1.6
50-51	505,007	200	203.1	(51.8)	261,436	198	201.4	(50.4)	243,571	202	205.0	(53.2)	-4	-3.6
52-53	454,406	202	204.8	(52.4)	236,260	198	200.7	(48.9)	218,146	206	209.1	(55.6)	-8	-8.4
54-55	450,635	203	205.6	(51.0)	235,215	198	200.5	(48.5)	215,420	208	211.2	(53.1)	-10	-10.7
56-57	390,911	203	206.1	(53.6)	198,187	197	200.0	(52.1)	192,724	209	212.4	(54.4)	-12	-12.4
58-59	347,132	202	205.3	(52.5)	174,366	196	198.7	(49.5)	172,766	209	212.0	(54.6)	-13	-13.3
60-61	384,271	201	204.6	(54.4)	189,991	194	197.4	(52.2)	194,280	209	211.7	(55.5)	-15	-14.4
62-63	361,062	201	204.2	(58.0)	174,138	193	196.3	(56.1)	186,924	208	211.5	(58.7)	-15	-15.2
64-65	323,187	200	203.6	(56.6)	155,662	192	195.4	(53.3)	167,525	208	211.2	(58.6)	-16	-15.9
66-67	280,786	200	203.0	(55.9)	132,790	191	194.4	(53.3)	147,996	207	210.7	(57.0)	-16	-16.3
68-69	239,383	199	202.6	(57.8)	111,671	190	193.5	(57.0)	127,712	207	210.5	(57.3)	-17	-17.0
70-71	185,926	199	202.6	(62.2)	84,436	189	193.4	(65.3)	101,490	207	210.3	(58.4)	-18	-17.0
72-73	142,558	198	201.8	(57.8)	61,930	188	191.6	(56.0)	80,628	206	209.7	(58.0)	-18	-18.1
74-75	112,889	198	201.9	(64.2)	48,880	187	191.3	(63.8)	64,009	206	210.0	(63.3)	-19	-18.7
76-77	82,237	197	200.6	(59.7)	35,677	186	189.2	(54.9)	46,560	205	209.3	(61.7)	-19	-20.1
78-79	57,463	196	198.6	(48.8)	25,735	185	187.6	(43.1)	31,728	204	207.6	(51.2)	-19	-20.0
80-81	42,346	194	198.1	(57.8)	18,802	184	186.9	(52.2)	23,544	203	207.0	(60.4)	-19	-20.1
82-83	26,015	193	197.0	(62.9)	11,568	182	186.4	(62.3)	14,447	201	205.5	(62.1)	-19	-19.2
84-85	14,482	191	195.8	(63.7)	6,482	180	184.9	(69.2)	8,000	201	204.6	(57.4)	-21	-19.7
86-99	16,013	190	193.3	(51.6)	6,602	179	182.3	(51.0)	9,411	198	201.0	(50.5)	-19	-18.7

SD, standard deviation.
[a] Concentration in men minus concentration in women.
To convert cholesterol from mg/dL to mmol/L, multiply by 0.02586.

Table S2. HRs[a] for death associated with fasting glucose, after adjustment for potential risk factors (Including numerical version of Figure 2).

sex	TC group, mg/dL	Person year	No. of death	Sex, and age adjusted[b] p-value	Sex, and age adjusted[b] HR (95% CI)	Fully adjusted[c] p-value	Fully adjusted[c] HR (95% CI)
Men and Women	<120	1,251,757	13,600	<0.001	2.26 (2.22-2.31)	<0.001	2.03 (1.99-2.07)
	120-129	1,929,661	13,287	<0.001	1.81 (1.77-1.84)	<0.001	1.68 (1.65-1.71)
	130-139	4,219,402	24,511	<0.001	1.59 (1.57-1.61)	<0.001	1.49 (1.47-1.52)
	140-149	6,754,049	34,398	<0.001	1.41 (1.39-1.43)	<0.001	1.35 (1.33-1.37)
	150-159	9,857,135	47,598	<0.001	1.29 (1.27-1.30)	<0.001	1.25 (1.23-1.26)
	160-169	12,552,009	58,289	<0.001	1.19 (1.18-1.21)	<0.001	1.17 (1.15-1.18)
	170-179	14,239,120	66,208	<0.001	1.12 (1.11-1.14)	<0.001	1.11 (1.09-1.12)
	180-189	14,964,456	69,918	<0.001	1.07 (1.06-1.09)	<0.001	1.06 (1.05-1.08)
	190-199	14,633,057	69,776	<0.001	1.05 (1.03-1.06)	<0.001	1.04 (1.03-1.06)
	200-209	12,952,005	64,340	<0.001	1.03 (1.02-1.04)	<0.001	1.03 (1.02-1.04)
	210-219	10,934,462	55,757	0.065	1.01 (1.00-1.02)	0.098	1.01 (1.00-1.02)
	220-229	8,960,734	46,623		1.00 (Reference)		1.00 (Reference)
	230-239	6,568,184	35,346	0.381	1.01 (0.99-1.02)	0.446	1.01 (0.99-1.02)
	240-249	4,799,485	26,936	0.063	1.01 (1.00-1.03)	0.208	1.01 (0.99-1.02)
	250-259	3,593,413	21,662	<0.001	1.06 (1.05-1.08)	<0.001	1.06 (1.04-1.07)
	260-269	2,353,108	14,499	<0.001	1.07 (1.05-1.09)	<0.001	1.05 (1.03-1.07)
	270-279	1,423,317	9,300	<0.001	1.13 (1.10-1.15)	<0.001	1.10 (1.07-1.12)
	≥280	2,915,081	22,375	<0.001	1.28 (1.26-1.30)	<0.001	1.23 (1.21-1.25)
Men	<120	723,347	11,239	<0.001	2.45 (2.40-2.51)	<0.001	2.14 (2.09-2.19)
	120-129	1,068,428	10,648	<0.001	1.91 (1.87-1.95)	<0.001	1.73 (1.69-1.77)
	130-139	2,328,158	19,185	<0.001	1.67 (1.64-1.70)	<0.001	1.53 (1.50-1.56)
	140-149	3,700,041	26,152	<0.001	1.47 (1.45-1.50)	<0.001	1.38 (1.36-1.41)
	150-159	5,432,347	35,199	<0.001	1.33 (1.31-1.35)	<0.001	1.27 (1.25-1.29)
	160-169	7,018,011	41,965	<0.001	1.23 (1.21-1.25)	<0.001	1.18 (1.17-1.20)
	170-179	8,070,894	46,155	<0.001	1.15 (1.13-1.16)	<0.001	1.12 (1.10-1.13)
	180-189	8,617,076	47,166	<0.001	1.09 (1.07-1.11)	<0.001	1.07 (1.06-1.09)
	190-199	8,568,492	45,737	<0.001	1.06 (1.04-1.07)	<0.001	1.05 (1.03-1.06)
	200-209	7,613,334	40,632	<0.001	1.03 (1.02-1.05)	<0.001	1.03 (1.01-1.04)
	210-219	6,413,884	33,878	0.210	1.01 (0.99-1.03)	0.386	1.01 (0.99-1.02)
	220-229	5,235,961	27,495		1.00 (Reference)		1.00 (Reference)
	230-239	3,805,814	20,099	0.528	1.01 (0.99-1.02)	0.559	1.01 (0.99-1.02)
	240-249	2,729,948	14,776	0.108	1.02 (1.00-1.04)	0.216	1.01 (0.99-1.03)

sex	TC group, mg/dL	Person year	No. of death	Sex, and age adjusted[b] p-value	HR (95% CI)		Fully adjusted[c] p-value	HR (95% CI)	
	250-259	2,012,779	11,438	<0.001	1.06	(1.04-1.09)	<0.001	1.05	(1.03-1.08)
	260-269	1,288,280	7,368	<0.001	1.07	(1.04-1.10)	<0.001	1.06	(1.03-1.08)
	270-279	768,371	4,699	<0.001	1.15	(1.12-1.19)	<0.001	1.12	(1.09-1.16)
	≥280	1,500,690	10,715	<0.001	1.33	(1.30-1.36)	<0.001	1.26	(1.23-1.29)
Women	<120	528,410	2,361	<0.001	1.83	(1.75-1.91)	<0.001	1.74	(1.66-1.81)
	120-129	861,232	2,639	<0.001	1.64	(1.58-1.71)	<0.001	1.60	(1.54-1.67)
	130-139	1,891,244	5,326	<0.001	1.49	(1.44-1.53)	<0.001	1.45	(1.41-1.49)
	140-149	3,054,008	8,246	<0.001	1.32	(1.29-1.36)	<0.001	1.30	(1.27-1.34)
	150-159	4,424,787	12,399	<0.001	1.24	(1.21-1.27)	<0.001	1.22	(1.20-1.25)
	160-169	5,533,997	16,324	<0.001	1.16	(1.13-1.18)	<0.001	1.15	(1.13-1.17)
	170-179	6,168,225	20,053	<0.001	1.11	(1.09-1.13)	<0.001	1.11	(1.08-1.13)
	180-189	6,347,380	22,752	<0.001	1.07	(1.05-1.09)	<0.001	1.07	(1.05-1.09)
	190-199	6,064,564	24,039	<0.001	1.04	(1.02-1.06)	<0.001	1.04	(1.03-1.07)
	200-209	5,338,670	23,708	<0.001	1.03	(1.01-1.05)	0.003	1.03	(1.01-1.05)
	210-219	4,520,578	21,879	0.065	1.02	(1.00-1.04)	0.064	1.02	(1.00-1.04)
	220-229	3,724,774	19,128		1.00	(Reference)		1.00	(Reference)
	230-239	2,762,370	15,247	0.381	1.00	(0.98-1.02)	0.797	1.00	(0.98-1.02)
	240-249	2,069,537	12,160	0.063	1.00	(0.98-1.03)	0.966	1.00	(0.98-1.02)
	250-259	1,580,634	10,224	<0.001	1.06	(1.03-1.08)	<0.001	1.05	(1.03-1.08)
	260-269	1,064,828	7,131	<0.001	1.06	(1.03-1.09)	0.001	1.05	(1.02-1.07)
	270-279	654,946	4,601	<0.001	1.09	(1.06-1.13)	<0.001	1.07	(1.03-1.10)
	≥280	1,414,390	11,660	<0.001	1.23	(1.20-1.26)	<0.001	1.18	(1.16-1.21)

CI, confidence interval; HR, hazard ratio; TC, total cholesterol

[a] HRs were calculated by Cox models stratified by age (baseline age, years: 18-24, 25-34, 35-44, 45-54, 55-64, 65-74, 75-84, 85-99), after adjustment for risk factors.

[b] Adjustement for age at baseline, and sex (when applicable)

[c] Adjustement for age at baseline, sex (when applicable), smoking status, alcohol use, physical activity, known history of heart disease, stroke, or cancer, body mass index, systolic blood pressure, and fasting glucose.

To convert cholesterol from mg/dL to mmol/L, multiply by 0.02586.

Table S3. HRs[a] for death associated with total cholesterol in men and women, according to age. (Including numerical version of Figure 3).

Age group, years	TC group, mg/dL	Men (n=7,292,064)					Women (n=5,522,942)				
		Person year	No. of death	CDR[b]	p-value	HR (95% CI)	Person year	No. of death	CDR[b]	p-value	HR (95% CI)
18-34	<120	303,032	278	92	<0.001	1.53 (1.33-1.76)	243,846	87	36	0.350	1.13 (0.87-1.47)
	120-129	511,449	403	79	<0.001	1.33 (1.18-1.50)	452,588	143	32	0.874	1.02 (0.81-1.28)
	130-139	1,090,672	748	69	0.004	1.16 (1.05-1.28)	958,414	298	31	0.983	1.00 (0.83-1.22)
	140-149	1,664,807	1,130	68	0.003	1.15 (1.05-1.26)	1,462,502	408	28	0.244	0.90 (0.74-1.08)
	150-159	2,287,566	1,467	64	0.084	1.08 (0.99-1.18)	1,933,275	542	28	0.230	0.90 (0.75-1.07)
	160-169	2,755,016	1,717	62	0.349	1.04 (0.96-1.13)	2,144,521	602	28	0.199	0.89 (0.75-1.06)
	170-179	2,902,400	1,814	62	0.396	1.04 (0.95-1.13)	2,068,039	621	30	0.543	0.95 (0.79-1.13)
	180-189	2,856,258	1,644	58	0.195	0.95 (0.87-1.03)	1,798,297	524	29	0.300	0.91 (0.76-1.09)
	190-199	2,660,488	1,692	64	0.424	1.03 (0.95-1.12)	1,452,647	388	27	0.046	0.83 (0.69-1.00)
	200-209	2,155,762	1,349	63	0.865	1.01 (0.92-1.10)	1,004,278	308	31	0.530	0.94 (0.78-1.14)
	210-219	1,693,293	1,039	61	0.559	0.97 (0.89-1.07)	693,825	232	33	0.907	1.01 (0.83-1.24)
	220-229	1,293,881	826	64		1.00 (Reference)	467,354	156	33		1.00 (Reference)
	230-239	898,251	630	70	0.136	1.08 (0.98-1.20)	281,607	87	31	0.516	0.92 (0.71-1.19)
	240-249	603,822	423	70	0.273	1.07 (0.95-1.20)	172,996	65	38	0.526	1.10 (0.82-1.47)
	250-259	429,928	316	74	0.149	1.10 (0.97-1.25)	112,227	34	30	0.507	0.88 (0.61-1.28)
	260-269	264,699	225	85	0.003	1.25 (1.08-1.45)	64,721	28	43	0.304	1.23 (0.83-1.85)
	270-279	154,923	129	83	0.053	1.20 (1.00-1.45)	37,995	18	47	0.216	1.36 (0.84-2.22)
	≥280	288,329	296	103	<0.001	1.44 (1.26-1.64)	89,389	46	51	0.032	1.43 (1.03-1.99)
35-44	<120	150,536	725	482	<0.001	3.13 (2.88-3.40)	128,381	146	114	<0.001	1.40 (1.16-1.67)
	120-129	224,297	683	305	<0.001	2.11 (1.94-2.30)	206,714	183	89	0.152	1.13 (0.96-1.33)
	130-139	528,158	1,313	249	<0.001	1.76 (1.65-1.89)	470,783	439	93	0.006	1.19 (1.05-1.35)
	140-149	897,321	1,846	206	<0.001	1.47 (1.39-1.57)	785,221	612	78	0.836	0.99 (0.88-1.11)
	150-159	1,391,295	2,590	186	<0.001	1.34 (1.27-1.42)	1,157,387	955	83	0.453	1.04 (0.94-1.15)
	160-169	1,884,628	3,174	168	<0.001	1.22 (1.16-1.29)	1,465,788	1,158	79	0.779	0.99 (0.89-1.09)
	170-179	2,266,431	3,729	165	<0.001	1.18 (1.12-1.24)	1,614,337	1,238	77	0.243	0.94 (0.85-1.04)
	180-189	2,506,509	3,981	159	<0.001	1.14 (1.08-1.20)	1,603,757	1,239	77	0.188	0.94 (0.85-1.03)
	190-199	2,549,803	3,916	154	<0.001	1.09 (1.04-1.15)	1,445,618	1,106	77	0.072	0.91 (0.83-1.01)
	200-209	2,318,389	3,535	152	0.006	1.08 (1.02-1.13)	1,182,447	915	77	0.059	0.91 (0.82-1.00)
	210-219	1,984,850	2,919	147	0.383	1.02 (0.97-1.08)	904,568	733	81	0.195	0.93 (0.84-1.04)
	220-229	1,640,599	2,379	145		1.00 (Reference)	666,631	591	89		1.00 (Reference)
	230-239	1,202,907	1,895	158	0.023	1.07 (1.01-1.14)	443,147	358	81	0.082	0.89 (0.78-1.02)
	240-249	869,950	1,324	152	0.496	1.02 (0.96-1.09)	292,766	264	90	0.760	0.98 (0.85-1.13)
	250-259	643,394	1,100	171	<0.001	1.13 (1.05-1.21)	200,016	201	100	0.486	1.06 (0.90-1.24)

Age group, years	TC group, mg/dL	Men (n=7,292,064)					Women (n=5,522,942)				
		Person year	No. of death	CDR[b]	p-value	HR (95% CI)	Person year	No. of death	CDR[b]	p-value	HR (95% CI)
	260-269	410,907	710	173	0.011	1.12 (1.03-1.21)	120,119	132	110	0.225	1.12 (0.93-1.36)
	270-279	250,640	470	188	<0.001	1.19 (1.08-1.32)	67,067	83	124	0.059	1.25 (0.99-1.57)
	≥280	484,058	1,146	237	<0.001	1.42 (1.32-1.52)	127,889	196	153	<0.001	1.48 (1.26-1.74)
45-54	<120	102,907	1,542	1,498	<0.001	3.39 (3.20-3.60)	76,271	213	279	<0.001	1.93 (1.67-2.23)
	120-129	136,771	1,347	985	<0.001	2.37 (2.23-2.52)	107,313	255	238	<0.001	1.72 (1.51-1.96)
	130-139	309,813	2,499	807	<0.001	2.01 (1.91-2.11)	250,425	522	208	<0.001	1.55 (1.40-1.71)
	140-149	518,279	3,265	630	<0.001	1.61 (1.54-1.69)	437,913	783	179	<0.001	1.33 (1.22-1.45)
	150-159	826,178	4,576	554	<0.001	1.45 (1.39-1.51)	717,250	1,192	166	<0.001	1.23 (1.14-1.33)
	160-169	1,154,223	5,627	488	<0.001	1.29 (1.24-1.35)	1,013,582	1,590	157	<0.001	1.15 (1.07-1.23)
	170-179	1,441,776	6,343	440	<0.001	1.18 (1.13-1.22)	1,273,313	1,937	152	0.004	1.10 (1.03-1.18)
	180-189	1,654,586	6,885	416	<0.001	1.12 (1.08-1.16)	1,453,665	2,161	149	0.090	1.06 (0.99-1.13)
	190-199	1,736,882	6,772	390	0.005	1.06 (1.02-1.10)	1,502,476	2,192	146	0.613	1.02 (0.95-1.08)
	200-209	1,653,108	6,179	374	0.564	1.01 (0.97-1.05)	1,436,978	2,128	148	0.736	1.01 (0.95-1.08)
	210-219	1,455,496	5,444	374	0.748	1.01 (0.97-1.05)	1,279,375	1,967	154	0.347	1.03 (0.97-1.10)
	220-229	1,243,405	4,616	371		1.00 (Reference)	1,094,889	1,665	152		1.00 (Reference)
	230-239	929,267	3,454	372	0.725	0.99 (0.95-1.04)	826,553	1,299	157	0.861	1.01 (0.94-1.08)
	240-249	687,740	2,563	373	0.642	0.99 (0.94-1.04)	629,500	1,003	159	0.942	1.00 (0.93-1.08)
	250-259	518,194	2,038	393	0.214	1.03 (0.98-1.09)	481,588	832	173	0.125	1.07 (0.98-1.16)
	260-269	344,059	1,289	375	0.293	0.97 (0.91-1.03)	325,387	545	167	0.826	1.01 (0.92-1.11)
	270-279	202,709	886	437	0.003	1.12 (1.04-1.20)	199,811	356	178	0.415	1.05 (0.94-1.18)
	≥280	409,263	2,141	523	<0.001	1.28 (1.21-1.34)	413,422	943	228	<0.001	1.29 (1.19-1.39)
55-64	<120	93,669	3,196	3,412	<0.001	2.66 (2.55-2.77)	45,155	502	1,112	<0.001	2.42 (2.20-2.65)
	120-129	111,297	2,746	2,467	<0.001	2.06 (1.97-2.15)	52,462	492	938	<0.001	2.10 (1.91-2.31)
	130-139	233,295	4,864	2,085	<0.001	1.77 (1.71-1.84)	119,125	858	720	<0.001	1.64 (1.52-1.76)
	140-149	370,775	6,413	1,730	<0.001	1.54 (1.49-1.59)	206,575	1,389	672	<0.001	1.53 (1.43-1.62)
	150-159	565,775	8,464	1,496	<0.001	1.35 (1.31-1.40)	352,304	2,132	605	<0.001	1.40 (1.33-1.47)
	160-169	765,792	10,235	1,337	<0.001	1.24 (1.20-1.28)	527,983	2,811	532	<0.001	1.24 (1.18-1.30)
	170-179	931,020	11,270	1,210	<0.001	1.14 (1.11-1.18)	709,592	3,529	497	<0.001	1.16 (1.11-1.21)
	180-189	1,040,344	11,731	1,128	<0.001	1.08 (1.05-1.11)	885,810	4,088	461	<0.001	1.08 (1.04-1.13)
	190-199	1,075,089	11,708	1,089	<0.001	1.06 (1.03-1.09)	990,752	4,498	454	0.001	1.07 (1.03-1.12)
	200-209	996,941	10,515	1,055	0.042	1.03 (1.00-1.06)	1,029,894	4,563	443	0.046	1.04 (1.00-1.09)
	210-219	867,135	8,910	1,028	0.369	1.01 (0.98-1.05)	993,073	4,277	431	0.592	1.01 (0.97-1.06)
	220-229	725,119	7,313	1,009		1.00 (Reference)	907,481	3,865	426		1.00 (Reference)
	230-239	535,409	5,380	1,005	0.940	1.00 (0.96-1.03)	742,031	3,019	407	0.068	0.96 (0.91-1.00)
	240-249	394,698	4,087	1,035	0.252	1.02 (0.98-1.06)	594,659	2,573	433	0.743	1.01 (0.96-1.06)

Age group, years	TC group, mg/dL	Men (n=7,292,064)					Women (n=5,522,942)				
		Person year	No. of death	CDR[b]	p-value	HR (95% CI)	Person year	No. of death	CDR[b]	p-value	HR (95% CI)
	250-259	293,694	3,159	1,076	0.009	1.06 (1.01-1.10)	481,307	2,151	447	0.179	1.04 (0.98-1.09)
	260-269	188,688	2,024	1,073	0.034	1.05 (1.00-1.11)	339,878	1,485	437	0.834	1.01 (0.95-1.07)
	270-279	112,346	1,255	1,117	0.004	1.09 (1.03-1.16)	214,995	1,007	468	0.092	1.06 (0.99-1.14)
	≥280	225,289	2,996	1,330	<0.001	1.25 (1.20-1.31)	475,490	2,623	552	<0.001	1.22 (1.16-1.28)
65-74	<120	58,893	3,669	6,230	<0.001	1.81 (1.74-1.88)	27,264	818	3,000	<0.001	1.88 (1.74-2.02)
	120-129	68,027	3,527	5,185	<0.001	1.54 (1.48-1.60)	33,862	897	2,649	<0.001	1.68 (1.57-1.80)
	130-139	134,291	6,217	4,630	<0.001	1.37 (1.33-1.42)	73,512	1,751	2,382	<0.001	1.51 (1.43-1.59)
	140-149	202,735	8,691	4,287	<0.001	1.30 (1.26-1.34)	128,720	2,720	2,113	<0.001	1.36 (1.31-1.43)
	150-159	294,436	11,541	3,920	<0.001	1.22 (1.19-1.26)	211,362	4,120	1,949	<0.001	1.25 (1.21-1.30)
	160-169	378,862	13,765	3,633	<0.001	1.15 (1.12-1.18)	307,739	5,459	1,774	<0.001	1.15 (1.11-1.19)
	170-179	440,573	14,938	3,391	<0.001	1.08 (1.06-1.11)	405,579	6,797	1,676	<0.001	1.10 (1.06-1.14)
	180-189	470,576	15,183	3,226	<0.001	1.05 (1.02-1.08)	492,988	8,035	1,630	<0.001	1.07 (1.04-1.11)
	190-199	462,377	14,479	3,131	0.023	1.03 (1.00-1.06)	549,204	8,786	1,600	<0.001	1.06 (1.02-1.09)
	200-209	417,255	12,851	3,080	0.182	1.02 (0.99-1.05)	559,797	8,848	1,581	0.002	1.05 (1.02-1.08)
	210-219	353,837	10,679	3,018	0.600	1.01 (0.98-1.04)	534,761	8,212	1,536	0.191	1.02 (0.99-1.05)
	220-229	286,088	8,524	2,980		1.00 (Reference)	486,988	7,325	1,504		1.00 (Reference)
	230-239	208,036	6,090	2,927	0.466	0.99 (0.96-1.02)	386,884	5,988	1,548	0.113	1.03 (0.99-1.06)
	240-249	151,259	4,634	3,064	0.058	1.04 (1.00-1.07)	314,853	4,816	1,530	0.383	1.02 (0.98-1.05)
	250-259	110,620	3,411	3,084	0.031	1.04 (1.00-1.09)	252,615	4,065	1,609	<0.001	1.08 (1.04-1.12)
	260-269	69,217	2,175	3,142	0.012	1.06 (1.01-1.11)	178,782	2,921	1,634	0.002	1.07 (1.02-1.12)
	270-279	41,365	1,399	3,382	<0.001	1.13 (1.07-1.20)	112,434	1,889	1,680	<0.001	1.10 (1.05-1.16)
	≥280	81,411	3,036	3,729	<0.001	1.23 (1.18-1.29)	255,780	4,718	1,845	<0.001	1.18 (1.14-1.23)
75-99	<120	14,310	1,829	12,781	<0.001	1.43 (1.36-1.52)	7,492	595	7,942	<0.001	1.32 (1.21-1.43)
	120-129	16,586	1,942	11,708	<0.001	1.31 (1.24-1.39)	8,292	669	8,068	<0.001	1.40 (1.29-1.51)
	130-139	31,929	3,544	11,100	<0.001	1.23 (1.18-1.29)	18,985	1,458	7,680	<0.001	1.34 (1.26-1.42)
	140-149	46,123	4,807	10,422	<0.001	1.17 (1.12-1.22)	33,078	2,334	7,056	<0.001	1.21 (1.15-1.27)
	150-159	67,097	6,561	9,778	<0.001	1.10 (1.06-1.15)	53,209	3,458	6,499	<0.001	1.13 (1.08-1.18)
	160-169	79,491	7,447	9,368	<0.001	1.07 (1.03-1.12)	74,384	4,704	6,324	<0.001	1.11 (1.07-1.15)
	170-179	88,693	8,061	9,089	0.025	1.04 (1.01-1.09)	97,366	5,931	6,091	<0.001	1.08 (1.05-1.12)
	180-189	88,804	7,742	8,718	0.321	1.02 (0.98-1.06)	112,863	6,705	5,941	0.003	1.05 (1.02-1.09)
	190-199	83,853	7,170	8,551	0.654	1.01 (0.97-1.05)	123,867	7,069	5,707	0.095	1.03 (0.99-1.07)
	200-209	71,879	6,203	8,630	0.153	1.03 (0.99-1.07)	125,277	6,946	5,545	0.731	1.01 (0.97-1.04)
	210-219	59,274	4,887	8,245	0.425	0.98 (0.94-1.03)	114,976	6,458	5,617	0.246	1.02 (0.99-1.06)
	220-229	46,869	3,837	8,187		1.00 (Reference)	101,430	5,526	5,448		1.00 (Reference)
	230-239	31,945	2,650	8,296	0.504	1.02 (0.97-1.07)	82,147	4,496	5,473	0.461	1.01 (0.98-1.06)

Age group, years	TC group, mg/dL	Men (n=7,292,064)					Women (n=5,522,942)				
		Person year	No. of death	CDR[b]	p-value	HR (95% CI)	Person year	No. of death	CDR[b]	p-value	HR (95% CI)
	240-249	22,481	1,745	7,762	0.111	0.96 (0.90-1.01)	64,763	3,439	5,310	0.418	0.98 (0.94-1.03)
	250-259	16,950	1,414	8,342	0.380	1.03 (0.97-1.09)	52,881	2,941	5,561	0.113	1.04 (0.99-1.08)
	260-269	10,709	945	8,824	0.033	1.08 (1.01-1.16)	35,941	2,020	5,620	0.046	1.05 (1.00-1.11)
	270-279	6,389	560	8,765	0.069	1.09 (0.99-1.19)	22,643	1,248	5,512	0.365	1.03 (0.97-1.09)
	≥280	12,340	1,100	8,914	0.005	1.10 (1.03-1.18)	52,420	3,134	5,979	<0.001	1.12 (1.07-1.17)

CDR, crude death rate per 100,000 person-years; CI, confidence interval; TC, total cholesterol; HR, hazard ratio

[a] HRs were calculated by Cox models stratified by age (baseline age, years: 18-24, 25-34, 35-44, 45-54, 55-64, 65-74, 75-84, 85-99), after adjustment for age at baseline, sex (when applicable), smoking status, alcohol use, physical activity, known history of heart disease, stroke, or cancer, body mass index, systolic blood pressure, and fasting glucose.

[b] per 100,000 person-years.

To convert cholesterol from mg/dL to mmol/L, multiply by 0.02586.

Table S4. HRs[a] for death associated with three categories of total cholesterol, according to age.

Age group, years	TC group, mg/dL	Men and women			Men			Women		
		No. of death	p-value	HR (95% CI)	No. of death	p-value	HR (95% CI)	No. of death	p-value	HR (95% CI)
18-99	<200	397,585		1.00 (Reference)	283,446		1.00 (Reference)	114,139		1.00 (Reference)
all ages	200-239	202,066	<0.001	0.86 (0.86-0.87)	122,104	<0.001	0.84 (0.84-0.85)	79,962	<0.001	0.89 (0.88-0.90)
combined	≥240	94,772	<0.001	0.92 (0.92-0.93)	48,996	<0.001	0.91 (0.90-0.91)	45,776	<0.001	0.94 (0.93-0.95)
18-34	<200	14,506		1.00 (Reference)	10,893		1.00 (Reference)	3,613		1.00 (Reference)
	200-239	4,627	0.044	0.97 (0.93-1.00)	3,844	0.013	0.95 (0.92-0.99)	783	0.138	1.06 (0.98-1.15)
	≥240	1,580	<0.001	1.13 (1.07-1.19)	1,389	<0.001	1.12 (1.06-1.19)	191	0.002	1.26 (1.09-1.46)
35-44	<200	29,033		1.00 (Reference)	21,957		1.00 (Reference)	7,076		1.00 (Reference)
	200-239	13,325	<0.001	0.85 (0.83-0.87)	10,728	<0.001	0.83 (0.81-0.85)	2,597	0.018	0.95 (0.90-0.99)
	≥240	5,626	<0.001	0.95 (0.92-0.98)	4,750	<0.001	0.92 (0.89-0.95)	876	<0.001	1.15 (1.07-1.23)
45-54	<200	49,701		1.00 (Reference)	38,856		1.00 (Reference)	10,845		1.00 (Reference)
	200-239	26,752	<0.001	0.80 (0.78-0.81)	19,693	<0.001	0.77 (0.76-0.78)	7,059	<0.001	0.89 (0.86-0.92)
	≥240	12,596	<0.001	0.85 (0.83-0.87)	8,917	<0.001	0.82 (0.80-0.84)	3,679	0.011	0.95 (0.92-0.99)
55-64	<200	90,926		1.00 (Reference)	70,627		1.00 (Reference)	20,299		1.00 (Reference)
	200-239	47,842	<0.001	0.81 (0.80-0.82)	32,118	<0.001	0.80 (0.79-0.81)	15,724	<0.001	0.83 (0.81-0.85)
	≥240	23,360	<0.001	0.87 (0.86-0.88)	13,521	<0.001	0.86 (0.85-0.88)	9,839	<0.001	0.88 (0.86-0.90)
65-74	<200	131,393		1.00 (Reference)	92,010		1.00 (Reference)	39,383		1.00 (Reference)
	200-239	68,517	<0.001	0.88 (0.87-0.89)	38,144	<0.001	0.87 (0.86-0.88)	30,373	<0.001	0.89 (0.88-0.90)
	≥240	33,064	<0.001	0.94 (0.93-0.96)	14,655	<0.001	0.94 (0.92-0.96)	18,409	<0.001	0.94 (0.93-0.96)
75-99	<200	82,026		1.00 (Reference)	49,103		1.00 (Reference)	32,923		1.00 (Reference)
	200-239	41,003	<0.001	0.92 (0.91-0.93)	17,577	<0.001	0.93 (0.91-0.94)	23,426	<0.001	0.92 (0.90-0.94)
	≥240	18,546	<0.001	0.95 (0.94-0.97)	5,764	<0.001	0.95 (0.92-0.97)	12,782	<0.001	0.95 (0.93-0.97)

CI, confidence interval; HR, hazard ratio; TC

[a] HRs were calculated by Cox models stratified by age (baseline age, years: 18-24, 25-34, 35-44, 45-54, 55-64, 65-74, 75-84, 85-99), after adjustement for age at baseline, sex (when applicable), known pre-existing diabetes, smoking status, alcohol use, physical activity, known history of heart disease, stroke, or cancer, body mass index, systolic blood pressure, and fasting glucose.

To convert cholesterol from mg/dL to mmol/L, multiply by 0.02586.
